# Subthreshold Diode Micropulse Laser Photocoagulation (SDM) as Invisible Retinal Phototherapy for Diabetic Macular Edema: A Review

**DOI:** 10.2174/157339912800840523

**Published:** 2012-07

**Authors:** Jeffrey K Luttrull, Giorgio Dorin

**Affiliations:** 1 3160 Telegraph Rd, Suite 230, Ventura, California 93003, USA; 2 Clinical Application Development, IRIDEX Corporation, 1212 Terra Bella Ave, Mountain View, California 94043, USA

**Keywords:** Subthreshold, invisible, diode laser, micropulse, photocoagulation, phototherapy, photostimulation, diabetes, diabetic retinopathy, diabetic macular edema.

## Abstract

**Purpose:**

To present the state-of-the-art of subthreshold diode laser micropulse photocoagulation (SDM) as invisible retinal phototherapy for diabetic macular edema (DME).

**Method:**

To review the role and evolution of retinal laser treatment for DME.

**Results:**

Thermal laser retinal photocoagulation has been the cornerstone of treatment for diabetic macular edema for over four decades. Throughout, laser induced retinal damage produced by conventional photocoagulation has been universally accepted as necessary to produce a therapeutic benefit, despite the inherent risks, adverse effects and limitations of thermally destructive treatment. Recently, SDM, performed as invisible retinal phototherapy for DME, has been found to be effective in the absence of any retinal damage or adverse effect, fundamentally altering our understanding of laser treatment for retinal disease.

**Summary:**

The discovery of clinically effective and harmless SDM treatment for DME offers exciting new information that will improve our understanding of laser treatment for retinal disease, expand treatment indications, and improve patient outcomes.

## INTRODUCTION

Information theory states that the amount of information available from a particular event is directly proportional to the unlikelihood of that event occurring [1]. It can be argued that selective burning of the retina with lasers has represented the single most important advance in the treatment of retinal disease. That iatrogenic retinal damage is necessary for effective laser treatment of retinal vascular disease has been universally accepted for almost 5 decades, and remains the prevailing notion [2]. Given the longstanding uniformity of opinion regarding the essential role of laser-induced retinal damage, the finding that retinal laser treatment that does not cause any laser-induced retinal damage can be at least effective as conventional retinal photocoagulation is unexpected, and thus powerfully informative [3-10]. If using lasers to burn the retina represents the seminal advance in the treatment of retinal disease, then learning that those retinal burns are unnecessary may constitute another significant advance, one that may fundamentally alter our understanding of retinal laser treatment for retinal vascular disease and the disease process.

We will examine this invisible retinal phototherapy, currently epitomized by subthreshold diode micropulse (SDM) laser treatment, in the treatment of diabetic macular edema (DME). 

### Epidemiology of DME

DME is the most common cause of visual loss in persons under 50 years of age in the developed world. Diabetes mellitus (DM), the cause of diabetic retinopathy and thus DME, is increasing in incidence and prevalence worldwide, becoming epidemic not only in the developed world, but in the developing world as well. Diabetic retinopathy may begin to appear in persons with type I (insulin-dependent) DM within 3 – 5 years of disease onset. By 20 years, nearly 100% will have some degree of diabetic retinopathy. While the retinal complications of diabetes can be attenuated by long-term intensive glycemic control, the prevalence of diabetic retinopathy increases with duration of disease. By 10 years, between 14 – 25% of patients will have DME [11-13]. Untreated, patients with “clinically significant” DME have a 32% 3-year risk of potentially disabling “moderate” visual loss, defined as doubling of the visual angle [14]. In addition to individual disability, the social and economic costs of the global diabetes pandemic – and thus DME – can hardly be overestimated. 

### Conventional Thermal Macular Photocoagulation for DME

Until the advent of thermal retinal photocoagulation there was no generally effective treatment for diabetic retinopathy. Using photocoagulation to produce photothermal retinal burns as a therapeutic maneuver was prompted by the observation that the complications of diabetic retinopathy were often less severe in eyes with preexisting retinal scarring from other causes. Photocoagulation, an effective means of producing retinal scars, was initially directed at treatment of proliferative diabetic retinopathy, using first the xenon arc photocoagulator, followed by ruby pulsed-laser and argon continuous-wave lasers (CWL). Spurred by success treating proliferative diabetic retinopathy, thermal laser photocoagulation was soon adapted to treatment of DME as well [14, 15]. 

The Early Treatment of Diabetic Retinopathy Study (ETDRS) demonstrated the efficacy of argon laser macular photocoagulation in the treatment of DME. While a small percentage of treated eyes enjoyed improvement in visual acuity (VA) following treatment, the main clinical benefit of macular photocoagulation was to reduce by 50% the rate of moderate visual loss (≥ 15 letters measured on a logarithmic VA chart) in eyes with mild to moderate nonproliferative diabetic retinopathy and “clinically significant” DME [14].

**Fig. (1):**
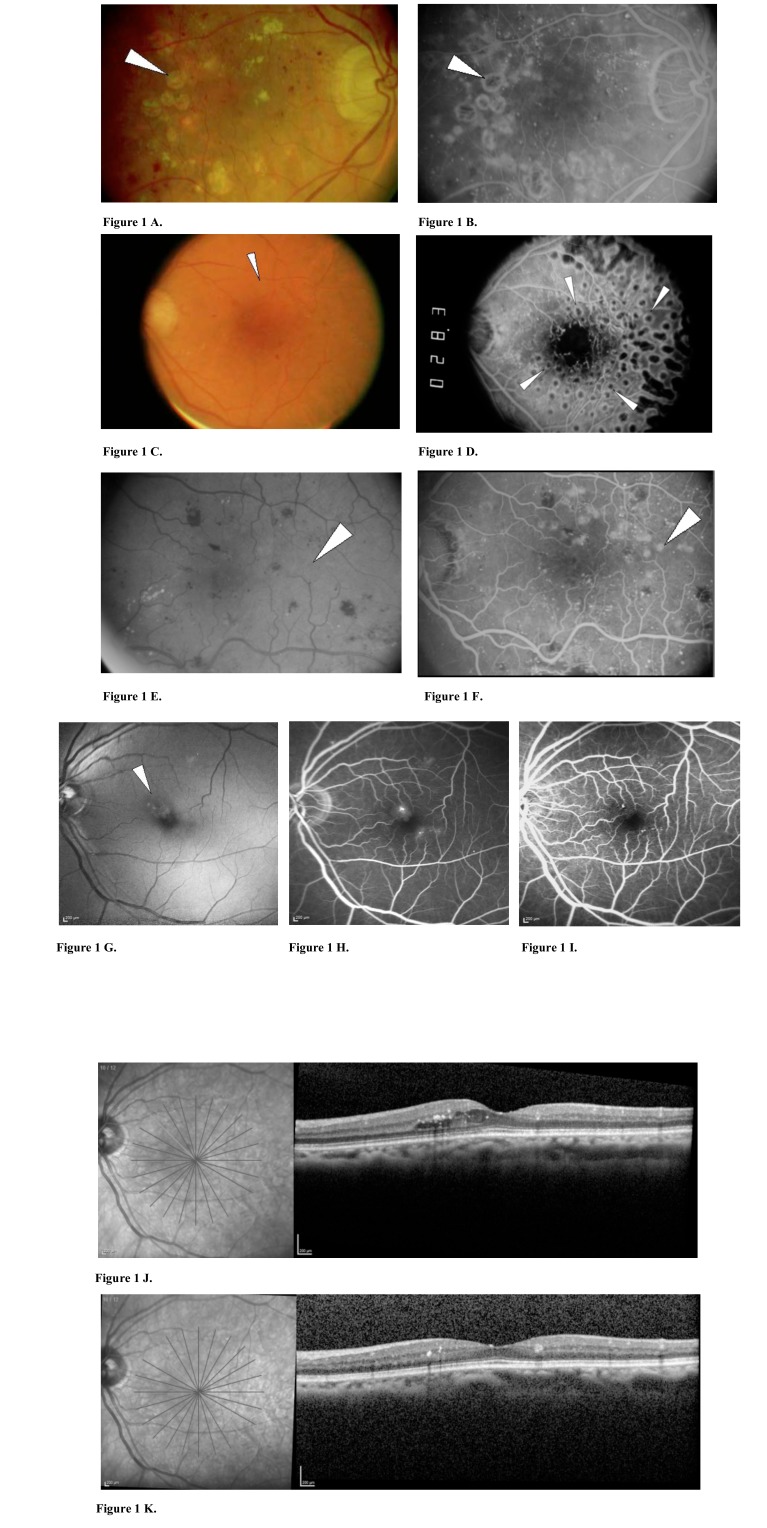
(**A**) Monochrome fundus photograph and (**B**) intravenous fundus fluorescein angiogram (FFA) of eye following conventional suprathreshold ETRDS-style macular photocoagulation for DME. Note full-thickness chorioretinal scars easily visible clinically and angiographically (arrows). (**C**) Monochrome fundus photograph and (**D**) FFA following “classical” continuous wave laser “subthreshold” macular photocoagulation. Note less obvious clinically visible retinal scars easily seen by FFA (arrows). (**E**) Color fundus photograph and (**F**) FFA following “clinical” “subthreshold” macular photocoagulation. Note absence of notable laser-induced retinal scarring on fundus photography that is easily seen by FFA (arrows). (**G**) Monochrome fundus photograph, (**H**) FFA pre- and (**I**) postoperatively and Spectral-domain OCT before (J) and 4 months after (K) “true” subthreshold macular laser treatment for DME (arrow) with SDM. Treatment consisted of 483 confluent SDM applications using a 131um retinal spot, 5% duty cycle, 0.3 second pulse duration, 0.9 Watt power placed throughout the area of macular thickening, including the nasal fovea, demonstrated by SD-OCT preoperatively. Note complete absence of laser-induced retinal damage with resolution of DME. Preoperative VA 20/30. Postoperative 20/25.

The method of laser treatment employed in the (ETDRS) came to set the technical standard for macular photocoagulation for DME. Full-thickness retinal laser burns in the areas of retinal pathology were created, visible at the time of treatment as white or light grey retinal lesions (“suprathreshold” retinal photocoagulation). With time, these lesions developed into focal areas of chorioretinal scarring and progressive atrophy. (Figs. **1A,B**) Although providing a clear advantage compared to no treatment, the inherent retinal damage and inflammation associated with suprathreshold macular photocoagulation (an iatrogenic multifocal chorioretinitis) was associated with a number of significant risks and adverse effects. These include early and late visual loss due to inadvertent foveal photocoagulation, incitement or aggravation of macular edema (ME) due to treatment-associated inflammation, pre- and sub-retinal fibrosis, choroidal neovascularization, visual field loss, loss of color vision, metamorphopsia, and progressive expansion of the laser scars into the fovea. Laser-induced retinal damage limited treatment density and repeatability. The ability to treat safely near the foveal center, the location of the most visually disabling form of DME, was limited as well [14 – 23]. Currently, the prevailing technique for laser treatment of DME is termed the “modified ETDRS” (mETDRS) photocoagulation technique. This technique, reflecting the preferred practice patterns of a panel of clinical researchers, preserves the general precepts of low-density patterned grid and focal (treatment of individual macular microaneurysms) ETDRS retinal photocoagulation for DME, but at lower suprathreshold treatment endpoint intensities [2].

In addition to establishing suprathreshold thermal macular photocoagulation as the standard of care, the ETDRS produced additional findings especially relevant to the evolution of laser treatment for DME. These include the observations that treatment risks and adverse effects increased with treatment intensity; that treatment effectiveness increased with treatment density; that the severity of macular thickening constituting DME and proximity to the central fovea were the primary risk factors for visual loss and best indicators of therapeutic response; that fundus fluorescein angiographic (FFA) leakage correlated poorly with macular thickening, VA, and treatment response; and that DME often responded to photocoagulation placed in a low-density grid-fashion throughout the area of macular swelling without direct laser treatment of macular microaneurysms. In recognition, there began steady movement toward reducing the intensity of the treatment endpoint. Longer laser wavelengths, such as the 647 nm krypton red and the 810 nm diode laser, not absorbed by macular chromophores, were employed to minimize damage to the neurosensory retina. Low-density grid-pattern applications of macular photocoagulations with less emphasis on higher-intensity focal photocoagulation of microaneurysms was increasingly employed and found to be effective [24-33]. However, laser-induced retinal damage indicated by a clinically visible retinal burn continued to be the desired endpoint – and thus limiting factor - of treatment [2].

### The Micropulsed Laser

In 1990, Pankratov reported development of a new laser modality designed to deliver laser energy in short pulses (“micropulses”) rather than as a continuous wave. Retinal lesion sizes produced by experimental thermal retinal photocoagulation with continuous wave (CW) and with pulsed laser energy were compared. For a same retinal spot size, pulse energy and pulse duration, the main determinant of retinal thermal lesion size from micropulsed laser photocoagulation was found to be the duty cycle (the frequency of the train of micropulses) and thus the length of the thermal relaxation time in between consecutive pulses. The lower the duty cycle and the longer the OFF time between pulses (lower repetition rate), the less heat build-up, resulting in less thermal retinal damage and smaller retinal laser lesions. By contrast, the higher the duty cycle, the more lesions produced by the micropulsed diode laser approximated those produced with a conventional CW laser. 

Using a longer wavelength near-infrared 810 nm diode laser, energy absorption by and thermal diffusion to the neurosensory retina could be minimized. By micropulsing the 810nm diode laser and reducing the frequency of laser micropulses by extending the “off-time” between micropulses (thus lowering the “duty cycle”) within the exposure envelope, higher laser energies and photothermal effects could be applied selectively to the retinal pigment epithelium (RPE), increasingly understood to be the source of potent extracellular factors acting as disease mediators, with less thermal retinal damage [34,35]. 

Beginning with Friberg in 1997 [36], a number of investigators reported use of the micropulsed diode laser (MPL) for treatment of DME. Treatment at that time was described as “invisible” as it was less clinically visible than traditional ETDRS-style treatment. However, MPL continued to be used to intentionally create retinal damage as an assumed prerequisite to therapeutically effective treatment, requiring adherence to traditional low-density treatment patterns. Thus, early use of the MPL remained limited in both safety and efficacy [36–46].

### Subthreshold Photocoagulation?

Precisely, “subthreshold” retinal photocoagulation is defined as retinal laser applications biomicroscopically invisible at the time of treatment. Unfortunately, the term has often been used to describe several different clinical scenarios reflecting widely varying degrees of laser-induced thermal retinal damage [25]. In general, use of the term “subthreshold” falls into 3 categories reflecting common usage and the historical and morphological evolution of reduced-intensity photocoagulation for retinal vascular disease toward truly invisible phototherapy [45].

“Classical” subthreshold photocoagulation describes the earliest attempts at laser intensity reduction using conventional CW argon, krypton, and diode lasers. As the retinal burns were notably less obvious than the white, full-thickness retinal burns produced according to ETDRS standards they were described as “subthreshold” or even “invisible” by comparison. Actually “threshold” (photocoagulation damage confined to the outer retina and thus less visible at the time of treatment) or even milder suprathreshold (full-thickness retinal photocoagulation generally easily visible at the time of treatment), the lesions of “classical” subthreshold photocoagulation were uniformly visible both clinically and by FFA at the time of treatment and thereafter [24 - 32, 36, 37] (Figs. **1A-D**).

“Clinical” subthreshold photocoagulation describes the next step in the evolution of laser-induced retinal damage reduction. This describes lower-intensity but persistently damaging retinal photocoagulation using either a micropulsed laser or short-pulsed (10 – 30ms) CW laser that better confines damage to the outer retina and RPE. In “clinical” subthreshold photocoagulation laser lesions may in fact be ophthalmoscopically invisible at the time of treatment. However, as laser-induced retinal damage remains the intended endpoint of treatment, laser lesions are produced which generally become increasingly clinically visible with time, and many, if not all, laser lesions can be seen by FFA, fundus autofluorescence photography (FAF), and / or Spectral-domain (SD) optical coherence tomography (OCT) at the time of treatment and thereafter [33, 36 – 45]. Since the original definition of “subthreshold” retinal photocoagulation was based upon the biomicroscopic appearance of the lesions at the time of treatment, “clinical” subthreshold photocoagulation is closest to the original definition of “subthreshold” [25] (Figs. **1E, F**).

“True” subthreshold photocoagulation expands the definition of “invisible by biomicroscopy” to include laser treatment non-discernable by any other known means such as FFA and newer high-resolution retinal imaging methods including FAF and SD-OCT. “True” subthreshold photocoagulation is therefore defined as laser treatment which produces absolutely no retinal damage detectable by any means at the time of treatment or anytime thereafter. Thus SDM, as a subtype of MPL, represents the fullest embodiment in the evolution of “subthreshold” laser treatment for retinal vascular disease (Figs. **1 G – K**). 

Inconsistency in use of the term “subthreshold” and transcendence of the original clinical definition by SDM suggests that newer descriptors such as “invisible retinal phototherapy” or “retinal photostimulation” may prove more meaningful and useful descriptors of SDM and future forms of truly invisible laser treatment for retinal disease [5] (Figs. **1 G –K**).

### Something New: “Low-Intensity / High-Density” SDM as Invisible Retinal Phototherapy for DME

In 2000 a pilot study was begun employing a new approach to laser treatment for retinal vascular disease. Using an 810nm diode laser in the micropulse emission mode, DME was treated for the first time with the intent of avoiding any laser-induced retinal damage (“low-intensity” treatment). Strict avoidance of laser-induced retinal injury facilitated a second fundamental change, that of complete and confluent treatment of all areas of macular thickening with numerous contiguous low-intensity laser applications (“high-density” treatment). No attempt was made to focally treat angiographically leaking microaneurysms (Figs. **1 G-K**). 

Reported in 2005, the clinical results of this new “low-intensity / high-density” MPL paradigm for DME, SDM, were found to be comparable to conventional photocoagulation but without any evidence of laser-induced retinal damage, complication, or adverse effect in any eye at the time of treatment, or any time thereafter by clinical examination, fundus photography, or FFA [4]. The assumptions guiding over four decades of laser treatment for the complications of retinal vascular disease were cast into doubt. Confirmed by subsequent studies and randomized clinical trials, it is now clear that laser treatment for DME can be performed which is at least as effective as conventional photocoagulation in the complete absence of laser-induced retinal damage [3, 5–10, 39]. A dramatic departure from longstanding universally held belief, the seeming unlikelihood of this finding offers a potential wealth of new information [1, 2]. 

### No Laser-induced Retinal Damage? How Does SDM Work?

Traditional theories developed to explain the therapeutic mechanism of conventional retinal photocoagulation proceeded from the assumption that laser-induced retinal damage was necessary to produce a beneficial therapeutic effect. The evidence supporting this belief was the circumstantial association with retinal burning and improved clinical outcomes. The therapeutic effects attributed to laser-induced thermal retinal destruction include reduced metabolic demand, debulking of diseased retina, increased intraocular oxygen tension and altered production vasoactive cytokines including vascular endothelial growth factor (VEGF) [2, 25, 33, 47]. But is this thermal retinal damage necessary? Does it account for the benefits of conventional laser treatment in the ways hypothesized? If so, does it offer a net therapeutic benefit - once all of the risks and adverse effects are fully considered - superior to non-damaging laser treatment? The results of SDM for DME suggest not. 

In the absence of retinal damage, how might SDM work? First, in the absence of laser-induced retinal damage there is no loss of functional retinal tissue and no inflammatory response to treatment. Adverse treatment effects are thus completely eliminated and functional retina preserved rather than sacrificed. The tissue destruction and subsequent inflammation inherent in conventional photocoagulation may explain the superior VA results seen in studies using drug therapy alone vs. drug therapy combined with conventional laser for treatment of DME [48]. 

Secondly, SDM spares the neurosensory retina and is selectively absorbed by the RPE. Current theories of the pathogenesis of retinal vascular disease especially implicate cytokines, potent extracellular vasoactive factors produced by the RPE, as important mediators of retinal vascular disease such as DME [50, 51]. SDM both selectively targets and avoids lethal heat build-up within the RPE (see “Dosimetry” below). With SDM the capacity for the treated RPE to participate in a therapeutic response is preserved and even enhanced rather than eliminated. 

Thirdly, low power red and near-infrared laser exposure is known to affect many cell types, particularly altering the behavior of cells in pathologic environments (such as diabetes) through a variety of intracellular photoacceptors. Cell function, and thus cytokine expression, is normalized and inflammation reduced [49]. This may explain in part the phenomenon of prompt subjective visual improvement often reported by patients following SDM for DME, recently corroborated by the observation of improved macular sensitivity measured by macular microperimetry in patients treated with SDM compared to conventional photocoagulation [4, 8]. The RPE elaborates many factors, known and unknown, that may mediate DME [50]. By normalizing function of viable RPE cells, SDM may induce changes in the expression of multiple factors physiologically as opposed to drug therapy that typically narrowly targets one or only a few post-cellular factors pharmacologically. Such laser-induced physiologic alteration of RPE cytokine expression may account for the slower onset and long lasting benefits observed following all modes of retinal laser treatment for DME [49-52].

Fourthly, it has been noted that the clinical effects of cytokines may follow a “U – shaped curve”, where small physiologic changes in cytokine production (denoted by the left side of the curve) may have large clinical effects comparable to high-dose (pharmacologic) therapy (denoted by the right side of the curve) [53]. Using sublethal laser exposures, SDM may be working on this left side of the curve where the treatment response may approximate more of an “on/off” phenomenon rather than dose-response. This might explain the clinical effectiveness of SDM observed at the lowest reported irradiances [3, 54]. Thus, consistent with both extensive clinical experience and *in-vitro* studies of laser-tissue interactions, increasing irradiance may simply increase the risk of thermal retinal damage without improving the therapeutic effect [5, 49]. 

**Fig. (2):**
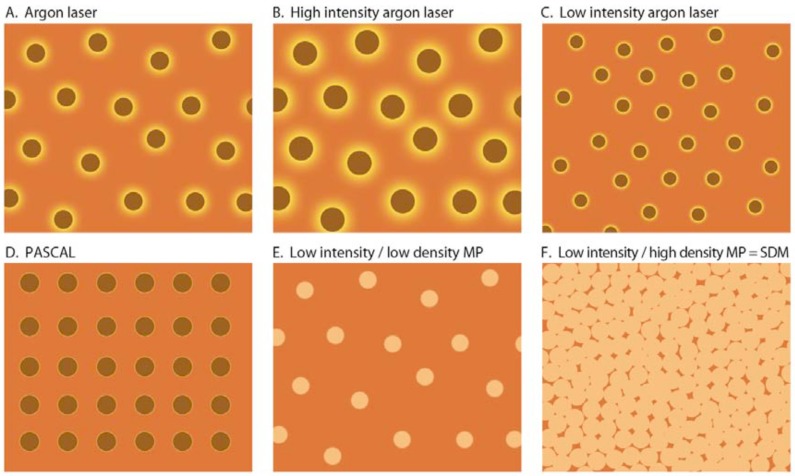
A-F. Graphic representation of the “Effective Surface Area” of various modes of retinal laser treatment for retinal vascular disease. Vermillion = Retina unaffected by laser treatment. Brown = Area of retina destroyed by laser and inactive with respect to ability to produce extracellular cytokines. Yellow = Area of retina affected by the laser but not destroyed, able to contribute to the therapeutic effects of laser treatment via laser-induced alteration / normalization of cytokine expression. PASCAL = pattern scanning laser. MP = diode micropulse laser. SDM = “High density / low-intensity” subthreshold / subvisible diode micropulsed laser.

Finally, high-density SDM amplifies all of the above effects by maximizing therapeutic recruitment of the RPE through the concept of “maximized effective surface area” [4-6, 54] (Fig. **2**). Laboratory studies suggest that the therapeutic alterations in RPE cytokine production elicited by conventional photocoagulation come from cells at the margins of traditional laser burns, affected but not killed by laser exposure [50, 51, 55, 56]. Thus, as illustrated in (Fig. **2A**), the therapeutic effect of conventional argon laser retinal photocoagulation may derive from a ring of affected but surviving RPE cells at the margin of each retinal burn. By increasing burn intensity, the width of this therapeutic ring would enlarge (Fig. **2B**), consistent with the observation that increased burn intensity is associated with an enhanced therapeutic effect, but hampered by increased loss of functional retina and inflammation. The converse would be expected with reduced intensity conventional argon laser photocoagulation. (Fig. **1C**) This may explain the inferior clinical results from lower-intensity / lower-density (“mild”) argon laser grid photocoagulation compared to higher-intensity / higher-density mETDRS treatment for DME [58]. Low-fluence photocoagulation with short-pulse (10- 30ms) CW lasers, such as the pattern scan laser (“PASCAL”, Topcon Medical Systems, Oakland, N.J., U.S.A.), produces minimal apical and lateral spread of laser photothermal tissue effects. (Fig. **2D**) Thus, despite complete ablation of the directly treated RPE and outer retina, the rim of therapeutically affected and surviving tissue is scant. Recent reports finding superiority of conventional argon laser panretinal photocoagulation over PASCAL for diabetic retinopathy may reflect this minimization of the effectively treated retinal surface area by short-pulse CWL [43, 58]. With low-intensity MP, however, all areas of the RPE exposed to laser irradiation are preserved, and available to contribute therapeutically. (Fig. **2E**) Permitted by the absence of tissue damage, SDM (low-intensity / high-density MP laser) is contiguously performed over all areas of retinal pathology (the “maximized effective surface area”), amplifying the therapeutic effect by maximizing therapeutic recruitment of the RPE (Fig. **2F**) A recent randomized clinical trial by Lavinsky, *et al.* demonstrated this phenomenon, finding SDM superior to both conventional mETDRS photocoagulation and low-density MPL in reducing macular thickening and improving VA in DME [7].

Thus, while traditional theories of conventional retinal photocoagulation were driven by attempts to explain the necessity and benefits of thermal retinal destruction and subsequent chorioretinal scarring, SDM theory is consistent with clinical observations, the known cellular effects of low-power near-infrared lasers, and our current understanding of the pathophysiology of DME and other retinal vascular disease. It is likely that conventional photocoagulation and SDM work through a common mechanism: improved retinal function and therapeutic modulation of cytokine production elicited by laser-induced sub-lethal photothermal stress and other effects produced in viable RPE cells. This effect is produced indirectly by conventional photocoagulation by the decaying thermal diffusion in non targeted RPE cells surrounding the laser burn, while it is produced directly by SDM in all targeted RPE cells irradiated with SDM. Thus, the risks and adverse effects of any retinal-destructive photocoagulation for retinal vascular disease appear to be unnecessary, and thus undesirable, side-effects of effective laser treatment.

### Dosimetry of Invisible Retinal Phototherapy

Ophthalmologists have long used the intraoperative ophthalmoscopic appearance of the laser-induced retinal burn to titrate laser power to the desired endpoint intensity. In the absence of a visible endpoint at the time of treatment, how does one “dose” the laser? 

Because of the high treatment density employed by SDM and the ability to safely treat to the edge of and through the foveal center using correct treatment parameters (Fig. **1 G-I**), strict and absolutely reliable avoidance of laser-induced retinal damage is imperative. It is equally imperative to perform clinically effective treatment. To this end we have three independent and corroborating sources that inform us regarding optimal SDM treatment parameters.

In the pilot study of SDM, it was noted that the American National Standards Institute (ANSI) has developed standards for safe work-place laser exposure based on a combination of theoretical and empirical data [4, 59 – 62]. This is the ANSI “maximum permissible exposure” (MPE) safety level, set at approximately 1/10^th^ of the laser exposure level expected to produce biologic effects. At a laser exposure level of one times MPE (1x MPE) absolute safety would be expected and retinal exposure to laser radiation at this level would be expected to have no biologic effect. Based on ANSI data, a 50% risk of suffering a barely visible (threshold) retinal burn is generally encountered at 10x MPE for conventional CW laser exposure. For a low-duty cycle MP laser exposure of the same power the risk of threshold retinal burn is approximately 100x MPE. Thus, the therapeutic range – the interval between doing nothing at all and the 50% likelihood of producing a threshold retinal burn – for low-duty cycle MP laser irradiation is 10 times wider than for CW laser irradiation with the same energy. Safe and effective SDM treatment has been reported at laser exposure levels as low as 18x and as high as 55x MPE [3, 5, 6, 54]. 

Thus, using the ANSI xMPE laser exposure model, the therapeutic window for SDM, within which effective treatment without thermal tissue injury would be expected, is broad [5]. Laser exposures above 1x MPE would be expected to produce a biologic effect. Laser exposure sufficiently below the 50% risk of burn threshold would be expected to produce biologic effects without discernable thermal retinal damage. It is remarkable to note that, in the absence of precedent, the laser parameters empirically employed in the pilot study of SDM deliver laser exposures to the retina at 47x MPE, in middle of the therapeutic window for MP laser exposure. At this level one would predict the observed clinical outcomes: therapeutic effectiveness with no discernable retinal damage. 

The long-term safety of SDM has been reported examining the retinal burn risk determined by FAF and FFA in 252 eyes treated for DME and macular edema due to branch retinal vein occlusion followed as long as 10 years (median 47 months) postoperatively. Inadvertent retinal burns were noted in 7 eyes. All were all evident at the first postoperative visit and all burns occurred in eyes treated at a 10% or 15% duty cycle (P=0.0001). No laser-induced retina injury was found in any eye treated with 5% duty cycle, despite the use of equivalent laser exposure levels of approximately 50x MPE for both groups [5].

In this same report, computational tissue temperature models were used to examine the risk of lethal cell injury at the SDM laser parameters used clinically. These calculations corroborated both clinical experience and ANSI MPE predictions. SDM performed with a 5% duty cycle demonstrated adequate thermal rise at the level of the RPE cell to stimulate a biologic response, but remained far below the level expected to produce lethal cell injury even in darkly pigmented fundi. In contrast, use of a 10% duty cycle adjusted to deliver MPL at similar irradiance and xMPE levels significantly increased the risk of lethal cell injury, particularly in darker fundi. Echoing extensive clinical experience, even a small increase in duty cycle from 5 to 10%, doubles the energy and reduces the molecular thermal relaxation time between micropulses, increasing in the risk of lethal thermal retinal injury 10-fold [5]. Thus small increases in MPL duty cycle significantly narrow therapeutic window, rapidly approaching the clinical behavior of continuous wave lasers. This effect is illustrated by the high incidence of laser-induced retinal lesions reported in all studies of MPL using visible test-burn titration algorithms and MPL duty cycles higher than 5% (“clinical” subthreshold retinal photocoagulation) [33, 36-45] (Figs. **1 E, F**).

Power limitations in diode lasers employed thus far require fairly long exposure duration (for example, 0.30 seconds) for SDM. The longer the laser exposure, the more important the center-spot heat dissipating facility toward the unexposed tissue at the margins of the laser spot and toward the underlying choriocapillaris, favoring use of small retinal laser spot sizes for SDM. This is illustrated by one study of use of a large-spot CW diode laser for subthreshold transpupillary thermotherapy treatment of DME which was abandoned following catastrophic visual loss in one study eye resulting from macular infarction. This was attributed to uneven heat distribution and insufficient heat dissipation within the large retinal laser spot [63]. 

Synthesizing the above information we can derive rational and evidence-based recommendations for SDM laser parameters expected to be both clinically effective and strictly and reliably safe, despite absence of a visible treatment endpoint or laser-induced retinal damage. Key parameters appear to be the following: a) a low (5% or less) duty cycle; b) a small spot size to minimize heat accumulation, assure uniform heat distribution within a given laser spot, and maximize heat dissipation; c) sufficient power to produce retinal laser exposures of between approximately 18x – 55x MPE producing an RPE temperature rise of 7 – 14⁰C; and d) high-density treatment of the pathologic retina with contiguous laser spots to maximize the therapeutic benefit by maximizing the effectively treated area. These are the essential ingredients of “low-intensity / high-density” SDM as invisible retinal phototherapy for DME. The agreement between ANSI MPE predictions, computational tissue temperature modeling and extensive clinical observations thus provides rational bases and useful tools for evaluating invisible retinal phototherapy protocols [64].

In another departure from conventional retinal photocoagulation, the wide therapeutic window of SDM and physical characteristics of the 810nm diode laser allow use of a single set of safe and effective laser parameters in all eyes with DME (“One size fits all”). (5) The 810nm diode laser is minimally absorbed and negligibly scattered by intraretinal blood, cataract, vitreous hemorrhage and even severely edematous neurosensory retina. Differences in fundus coloration result primarily from differences in choroid pigmentation, and less with variation in the target RPE. Treatment with SDM is thus simplified, requiring no adjustment in laser parameters for variations in macular thickening, intraretinal hemorrhage, media opacity or fundus pigmentation, reducing the risk of error.

We caution that the preceding analysis of SDM dosimetry is based on extensive clinical experience using the 810nm diode laser. Micropulse emission has recently become available in lasers with shorter wavelengths, such as 577nm yellow and 532nm green. The higher energies and different tissue absorption characteristics of shorter wavelength lasers may increase retinal burn risk, effectively narrowing the therapeutic window. In addition, shorter 532 and 577 nm wavelengths are more scattered by opaque ocular media,, retinal hemorrhage and macular edema, potentially limiting usefulness and increasing the risk of retinal damage in certain clinical settings. Although these wavelengths are effective in the closure of microaneurysms, such focal treatment is generally unnecessary, requiring higher laser irradiances that increase the risk of thermal retinal damage and is not a strategy of SDM (Figs. **1 G-K**). The parameters required for safe and effective SDM at wavelengths shorter than 810nm are currently under investigation.

### Implications for Use of SDM as Invisible Retinal Phototherapy for DME

The choice and administration of any medical intervention is informed by weighing the potential benefits vs. the possible risks and expected adverse effects of treatment. How then do we receive a treatment like SDM that appears comparably effective with conventional laser treatment, but is harmless, without risk or adverse treatment effect?

The diagnosis of “clinically significant” DME in the ETDRS was based upon the biomicroscopic finding of macular thickening, which is difficult to detect until the retinal thickness is increased by roughly 30% [65-68]. In this setting, conventional photocoagulation was found to reduce the risk of visual loss. Due to the difficulty identifying DME not reaching this threshold by biomicroscopy and the risks of conventional photocoagulation, earlier treatment was not justified. Due to the retinal damage inherent in conventional photocoagulation treatment was limited in density and in proximity to the fovea, where the most visually disabling DME occurs [14]. 

With the advent of OCT and newer, higher resolution iterations such as SD-OCT, we are now able to detect the very earliest stages of DME when the macular thickening may be only a few microns. Unless constrained by treatment risks, we know that earlier treatment of chronic and progressive diseases generally improves clinical outcomes. Many patients with early DME may be asymptomatic, with excellent VA. The risks of conventional photocoagulation are generally unacceptable in such eyes. By the same token, while often effective short-term, injection of intraocular drugs is such eyes is not without risk, and thus problematic as well. Currently, treatment of “sub-clinically significant” DME would appear to be a niche uniquely suited to SDM. This constitutes the largest number of patients with diabetic macular disease, as eyes with more severe “clinically significant” DME constitute only a subgroup of eyes at the extremity of diabetic maculopathy. The unique safety prolife of SDM permits us to offer earlier treatment of DME, when it is more likely to prevent visual disability and irreversible visual loss to a greater number of patients [5] (Figs. **1 G-K**).

Because SDM is harmless and has been found to be effective in all degrees of severity of DME, SDM would appear to be the ideal first-choice treatment for DME. By the same token, in the absence of retinal damage SDM can be repeated as many times as necessary without risk, suggesting it as an ideal retreatment measure in eyes that have incomplete or lack of response to prior treatments, especially eyes with pre-existing macular damage due to previous conventional photocoagulation [6]. 

Modern pharmacologic therapy, particularly use of intravitreal anti-VEGF inhibitors and anti-inflammatory drugs such as steroids, has been a boon to the management of DME and other retinal vascular disease. The benefits of drug therapy, particularly on VA, can be significant compared to conventional photocoagulation, particularly in the short-term. However, the long-term benefit from drug therapy compared to even conventional laser for DME is questionable [2, 50-52]. Because drug effects tend to be of rapid onset but brief, while laser effects slower in onset but long lasting, use of medication and laser in combination may be a benefit to selected patients with DME, particularly those presenting with severe center-involving DME and poor VA. Combination therapy holds the potential for rapid visual rehabilitation with reduced need for continuing drug therapy [5, 69]. Because the tissue damage and inflammation caused by conventional photocoagulation may be particularly detrimental in such eyes, SDM (also permitting safe transfoveal treatment) may be ideally suited for the treatment of center-involving ME alone or in combination with drugs [5, 48, 52] (Figs. **1 G-K**).

### The Future of Invisible Retinal Phototherapy for DME and Other Retinal Vascular Disease

The two most significant changes offered by invisible retinal phototherapy are the expansion of treatment indications permitted by the unique safety profile of SDM, and the new insights to be gained from the information that laser-induced retinal damage is not necessary to perform clinically efficacious retinal laser treatment for retinal vascular disease such as DME. 

With regard to the expansion of indications, we have discussed above the unique place SDM may have in treating sub-clinically significant DME diagnosed early, prior to symptomatic and irreversible visual loss, by new high-resolution imaging techniques such as SD-OCT. What of application to other retinal disorders? As of this writing SDM has been reported effective in the treatment of ME due to branch retinal vein occlusion, central serous chorioretinopathy, and proliferative diabetic retinopathy [54, 70-73]. 

In the pilot study of SDM panretinal photocoagulation, SDM appeared to reduce the progression of severe non proliferative to proliferative diabetic retinopathy without complication or adverse effect. These observations await confirmation by other investigators. However, what might be the benefit of harmless early intervention to reduce the risk and rate of progression of diabetic retinopathy? What of application to other forms of retinal vascular disease? If low-power laser exposure reduces inflammation and improves the “health” of the RPE, might SDM reduce progression and the risk of visual loss due to age-related macular degeneration? [49, 74].

As stated at the outset, information theory indicates that the very unlikelihood of SDM as effective treatment for DME opens the door to vast new opportunities for improvement of our understanding of retinal disease and the effects of laser - retinal interactions. Characterization of the products and pathways of the various therapeutically advantageous cytokines and other factors and effects elicited by invisible retinal phototherapy may lead to new treatment strategies and pharmacologic therapies to improve our ability to manage and even prevent retinal disease in entirely new ways. Knowledge that we no longer need to destroy our study subjects – RPE cells – enhances our ability to examine, detect and characterize these laser-induced cellular changes both *in vitro* and in vivo. SDM allows us to ask better, more informed questions, increasing the likelihood of better, more informative and useful answers. 

While SDM produces clinical effects akin to pharmacologic therapy, the slower onset and lasting effect suggest that the effects of SDM, like conventional photocoagulation, are mediated by secondary laser-induced changes in RPE cellular function [49]. Progressive improvement in retinal vascular macular edema is commonly observed for several years after a single session of SDM treatment [44, 70]. At the same time, recurrence of DME has been observed in some eyes, suggesting that the initially successful therapeutic effect of SDM had seemingly “worn off” [6, 44, 75, 76]. Why might SDM appear to work definitively in some eyes, but have a transient effect in others? How do we explain the finding of an apparent additive effect of repeat SDM treatment in some eyes with residual DME following SDM initial treatment? [6]. What is the role of SDM in conjunction with drug therapy, and how might combination therapy be optimized? We have discussed the practical complementarities of laser and drug therapy in eyes with center-involving DME and poor VA. However, if SDM is eliciting changes such as alteration of cellular cytokines production, and the elaboration of such factors is often regulated by local cellular feed-back mechanisms, can the nature and /or timing of drug therapy inhibit rather than compliment the effect of retinal laser treatment? If we use SDM to induce the RPE to physiologically produce less VEGF (among other possible changes), what might be the effect of temporally proximate pharmacologic introduction of vast quantities (2.0mg) of ranibizumab into the vitreous cavity? Could such pharmacologic therapy potentially cancel-out the effect of laser photostimulation *via* cellular feedback mechanisms? Combining therapies can lead to inhibition (a net effect less than the sum of its parts), complementarity (a net effect equal to the sum of its parts), or synergy (the ideal; a net effect greater than the sum of its parts). How can we avoid the former, and achieve the latter? 

Finally, the high-density treatment paradigm of SDM and use of small spot applications requires the application of many more laser spots than conventional photocoagulation. This is time consuming and can present a challenge to the busy clinician and the most cooperative patient. Automation is one answer to this problem. By virtue of its unique safety profile, SDM is ideally suited to incorporation in automated delivery systems and is an active area of development. 

## SUMMARY

SDM, as invisible retinal laser phototherapy, has been found to be effective in the treatment of DME. Upending nearly a half-century of universally held belief in the therapeutic necessity of laser-induced retinal damage, SDM offers exciting new information that will improve our understanding of retinal laser effects and the pathophysiology of retinal diseases, expand retinal laser treatment indications, ease case management and improve patient outcomes. A field in its infancy, the opportunities and possibilities for future research remain vast, exciting, and important. 

## References

[1]  Cover TM, Thomas JA Elements of information theory. Copyright
1991 John Wiley & Sons, Inc. Print ISBN 0-471-06259-6 Online
ISBN 0-471-20061-1.

[2]  Shah AM, Bressler NM, Jampol LM (2011). Does laser still have a role in
the management of retinal vascular and neovascular disease?. Am J
Ophthalmol.

[3]  Laursen ML, Moeller F, Sander B (2004). Subthreshold micropulse
diode laser treatment in diabetic macular oedema. Br J Ophthalmol.

[4]  Luttrull JK, Musch DC, Mainster MA (2005). Subthreshold diode micro-pulse
photocoagulation for the treatment of clinically significant
diabetic macular oedema. Br J Ophthalmol.

[5]  Luttrull JK, Sramek C, Palanker D, Spink CJ, Musch DC (2012). Long-term
safety, high-resolution imaging, and tissue temperature modeling
of subvisible diode micropulse photocoagulation for retinovascular
macular edema. Retina.

[6]  Luttrull JK, Spink CA (2006). Serial optical coherence tomography of
subthreshold diode micropulse photocoagulation for diabetic macular
edema. Ophthalmic Surg Lasers Imaging.

[7]  Lavinsky D, Cardillo JA, Melo LA, Dare A, Farah ME, Belfort R (2011). Randomized clinical trial evaluating mETDRS versus normal
or high-density micropulse photocoagulation for diabetic macular
edema. Invest Ophthalmol Vis Sci.

[8]  Vujosevic S, Bottega E, Casciano M, Pilotto E, Convento E, Midena E (2010). Microperimetry and fundus autofluorescence in diabetic
macular edema. Subthreshold micropulse diode laser versus modified
Early Treatment Diabetic Retinopathy Study Laser photocoagulation. Retina.

[9]  Venkatesh P, Ramanjulu R, Azad R, Vohra R, Garg S (2011). Subthreshold
micropulse diode laser and double frequency neodymium:
YAG laser in treatment of diabetic macular edema: a prospective,
randomized study using multifocal electroretinography. Photomed
Laser Surg.

[10]  Ohkoshi K, Yamaguchi T (2010). Subthreshold micropulse diode laser
photocoagulation for diabetic macular edema in Japanese patients. Am J Ophthalmol.

[11] DCCT Research Group (1993). The effect of intensive treatment of diabetes
in the development and progression of long-term complications
in insulin-dependent diabetes. N Engl J Med.

[12]  Klein R, Klein BE, Moss SE (1995). The Wisconsin epidemiologic
study of diabetic retinopathy. XV. The long-term incidence of
macular edema. Ophthalmology.

[13] Centers for Disease Control (www.cdc.gov/diabetes/news/docs/dpp.htm).

[14] Early Treatment Diabetic Retinopathy Study Research Group (1985). Photocoagulation for diabetic macular edema. ETDRS report no. 1. Arch Ophthalmol.

[15]  Wetzig P, Jepson C (1963). Treatment of diabetic retinopathy by light
coagulation: a preliminary study. Br J Ophthalmol.

[16]  Beetham W, Aiello L, Balodimos M (1970). Ruby laser photocoagulation
of early diabetic neovascular retinopathy: preliminary report
of a long-term controlled study. Arch Ophthalmol.

[17]  Schatz H, Madeira D, McDonald HR (1991). Progressive enlargement
of laser scars following grid laser photocoagulation for diffuse
diabetic macular edema. Arch Ophthalmol.

[18]  Morgan CM, Schatz H (1989). Atrophic creep of the retinal pigment epithelium
after focal macular photocoagulation. Ophthalmology.

[19] Early Treatment Diabetic Retinopathy Study Research Group (1995). Focal photocoagulation treatment of diabetic macular edema. Relationship
of treatment effect to fluorescein angiographic and other
retinal characteristics at baseline: ETDRS report no 19. Arch Ophthalmol.

[20]  Lewen RM (1988). Subretinal neovascularization complicating laser photocoagulation
of diabetic maculopathy. Ophthalmic Surg.

[21]  Lewis H, Schachat AP, Haimann MH (1990). Choroidal neovascularization
after laser photocoagulation for diabetic macular edema. Ophthalmology.

[22]  Guyer DR, D'Amico DJ, Smith CW (1992). Subretinal fibrosis after laser
photocoagulation for diabetic macular edema. Am J Ophthalmol.

[23]  Rutledge BK, Wallow IH, Poulsen GL (1993). Sub-pigment epithelial
membranes after photocoagulation for diabetic macular edema. Arch Ophthalmol.

[24]  Striph GG, Hart WM, Olk RJ (1988). Modified grid laser photocoagulation
for diabetic macular edema. The effect on the central visual
field. Ophthalmology.

[25]  Mainster MA (1999). Decreasing retinal photocoagulation damage: principles
and techniques. Sem Ophthalmol.

[26]  Olk RJ (1986). Modified grid argon (blue-green) laser photocoagulation
for diffuse diabetic macular edema. Ophthalmology.

[27]  Olk RJ (1990). Argon green (514 nm) versus krypton red (647 nm) modified
grid laser photocoagulation for diffuse diabetic macular
edema. Ophthalmology.

[28]  Lee CM, Olk RJ (1991). Modified grid laser photocoagulation for diffuse
diabetic macular edema. Long-term visual results. Ophthalmology.

[29]  Akduman L, Olk RJ (1997). Diode laser (810 nm) versus argon green (514
nm) modified grid photocoagulation for diffuse diabetic macular
edema. Ophthalmology.

[30]  Akduman L, Olk RJ (1999). Subthreshold (subvisible) modified grid diode
laser photocoagulation in diffuse diabetic macular edema (DDME). Ophthalmic Surg Lasers.

[31]  Roider J, Brinkmann R, Wirbelauer C (2000). Subthreshold (retinal
pigment epithelium) photocoagulation in macular diseases: a pilot
study. Br J Ophthalmol.

[32]  Olk RJ, Akduman L (2001). Minimal intensity diode laser (810 nanometer)
photocoagulation (MIP) for diffuse diabetic macular edema
(DDME). Sem Ophthalmol.

[33]  Blumenkranz MS (2010). Optimal current and future treatments for diabetic
macular oedema. Eye.

[34]  Pankratov MM (1990). Pulsed delivery of laser energy in experimental
thermal retinal photocoagulation. Proc Soc Photo-Optical Instrum
Eng.

[35]  Dorin G (2003). Subthreshold and micropulse photocoagulation. Sem
Opthalmol.

[36]  Friberg TR, Karatza EC (1997). The treatment of macular disease using a
micropulsed and continuous wave 810-nm diode laser. Ophthalmology.

[37]  Moorman CM, Hamilton AM (1999). Clinical applications of the Micro-Pulse diode laser. Eye.

[38]  Nakamura Y, Mitamura Y, Ogata K (2010). Functional and morphological
changes of macula after subthreshold micropulse diode laser
photocoagulation for diabetic macular oedema. Eye.

[39]  Figueira J, Khan J, Nunes S (2009). Prospective randomised controlled
trial comparing sub-threshold micropulse diode laser photocoagulation
and conventional green laser for clinically significant
diabetic macular oedema. Br J Ophthalmol.

[40]  Takatsuna Y, Yamamoto S, Nakamura Y (2011). Long-term therapeutic
efficacy of the subthreshold micropulse diode laser photocoagulation
for diabetic macular edema. Jpn J Ophthalmol.

[41]  Stanga PE, Reck AC, Hamilton AM (1999). Micropulse laser in the treatment
of diabetic macular edema. Semin Ophthalmol.

[42]  Nagpal M, Marlecha S, Nagpal K (2010). Comparison of laser photocoagulation
for diabetic retinopathy using 532-nm standard laser versus
multispot pattern scan laser. Retina.

[43]  Chappelow AV, Tan K, Waheed NK (2012). Panretinal photocoagulation
for proliferative diabetic retinopathy: Pattern Scan Laser versus
argon laser. Am J Ophthalmol.

[44]  Luttrull JK, Boyd S, Cortez R, Sabates N (2010). Subthreshold retinal photocoagulation for diabetic
retinopathy. Retinal and Vitreoretinal
Diseases and Surgery. Chapter 7. ISBN: 98-9962-678-
23-6. Jaypee- Highlighlights Medical Publishers, Inc, Panama, Rep.
of Panama.

[45]  Bhagat N, Zarbin M (2004). Use of subthreshold diode micropulse laser
for treating diabetic macular edema. Contemp Ophthalmol.

[46]  Friberg TR (1999). Subthreshold (invisible) modified grid diode laser
photocoagulation and diffuse diabetic macular edema. Ophthalmic
Surg Lasers.

[47]  Lange CA, Stavrakas P, Luhmann UF (2011). Intraocular oxygen
distribution in advanced proliferative diabetic retinopathy. Am J
Ophthalmol.

[48]  Mitchell P, Bandello F, Schmidt-Erfurth U, RESTORE study
group (2011). The RESTORE study: ranibizumab monotherapy or combined
with laser versus laser monotherapy for diabetic macular
edema. Ophthalmology.

[49]  Gao X, Xing D (2009). Molecular mechanisms of cell proliferation induced
by low power laser irradiation. J Biomed Sci.

[50]  Duh EJ, Yang HS, Suzuma I, Miyagi M (2002). Pigment epithelium-derived
factor suppresses ischemia-induced retinal neovascularization
and VEGF-induced migration and growth. Invest Ophthalmol
Vis Sci.

[51]  Sohn HJ, Han DH, Kim IT (2011). Changes in aqueous concentrations
of various cytokines after intravitreal triamcinolone versus
bevacizumab for diabetic macular edema. Am J Ophthalmol.

[52]  Masoud S, Heidari GK, Alireza R (2012). Two-year results of a
randomized trial of intravitreal bevacizumab alone or combined
with triamcinolone versus laser in diabetic macular edema. Retina.

[53]  Spinas GA, Mandrup-Poulsen T, Molvig J (1986). Low concentrations
of interleukin-1 stimulate and high concentrations inhibit insulin release from isolated rat islets of Langerhans. Acta Endocrinol (Copenhagen).

[54]  Luttrull JK, Spink CJ, Musch DA (2008). Subthreshold diode micropulse
panretinal photocoagulation for proliferative diabetic retinopathy. Eye.

[55]  Flaxel C, Bradle J, Acott T (2007). Retinal pigment epithelium produces
matrix metalloproteinases after laser treatment. Retina.

[56]  Miura Y, Treumer F, Klettner A (2010). VEGF and PEDF secretions
over time following various laser irradiations on an RPE organ culture. Invest Ophthalmol Vis Sci.

[57] Fong DS, Strauber SF, Aiello LP, Writing Committee for the Diabetic Retinopathy Clinical Research
Network (2007). Comparison of
the modified Early Treatment Diabetic Retinopathy Study and mild
macular grid laser photocoagulation strategies for diabetic macular
edema. Arch Ophthalmol.

[58]  Palanker D, Lavinsky D, Blumenkranz MS, Marcellino G (2011). The
impact of pulse duration and burn grade on size of retinal photocoagulation
lesion: Implications for pattern density. Retina.

[59] American National Standards Institute (2000). American national standard
for the safe use of lasers, ANSI Z136.1-2000.

[60]  Sliney DH, Wolbarsht ML (1980). Safety with lasers and other optical
sources: a comprehensive handbook.

[61]  Sliney DH, Mellerio J, Gabel VP (2002). What is the meaning of
threshold in laser injury experiments? Implications for human exposure
limits. Health Phys.

[62]  Mainster MA, Turner PL, Ryan SJ, Ogden TE, Hinton DR, 
Schachat AP (2004). Retinal injuries from light: mechanisms,
hazards and prevention. Retina.

[63]  Squirrell DM, Stewart AW, Joondeph BC (2008). Large-spot subthreshold
infrared laser to treat diabetic macular edema. Retina.

[64]  Sramek C, Mackanos M, Spitler R (2011). Non-damaging retinal
phototherapy: dynamic range of heat shock protein expression. Invest
Ophthalmol Vis Sci.

[65]  Strom C, Sander B, Larsen N, Larsen M, Lund-Andersen H (2002). Diabetic
macular edema assessed with optical coherence tomography
and stereo fundus photography. Invest Ophthalmol Vis Sci.

[66]  Moreira RO, Trujillo FR, Meirelles RM, Ellinger VC, Zagury L (2001). Use of optical coherence tomography (OCT) and indirect ophthalmoscopy
in the diagnosis of macular edema in diabetic patients. Int
Ophthalmol.

[67]  Brown JC, Solomon SD, Bressler SB (2004). Detection of diabetic foveal
edema. Arch Ophthalmol.

[68]  Panozzo G, Gusson E, Parolini B (2003). Role of OCT in the diagnosis
and follow up of diabetic macular edema. Semin Ophthalmol.

[69]  Smiddy WE (2011). Economic considerations of macular edema therapies. Ophthalmology.

[70]  Parodi MB, Spasse S, Iacono P, Di Stefano G, Canziani T, Ravalico 
G (2006). Subthreshold grid laser treatment of macular edema secondary
to branch retinal vein occlusion with micropulse infrared
(810 nanometer) diode laser. Ophthalmology.

[71]  Parodi MB, Iacono P, Ravalico G (2008). Intravitreal triamcinolone acetonide
combined with subthreshold grid laser treatment for macular
edema in branch retinal vein occlusion: A pilot study. Br J Ophthalmol.

[72]  Lanzetta P, Furlan F, Morgante L, Verritti D, Bandello F (2008). Nonvisible
subthreshold micropulse diode laser (810 nm) treatment of central
serous chorioretinopathy. A pilot study. Eur J Ophthalmol.

[73]  Koss MJ, Berger I, Koch FH (2012). Subthreshold diode laser micropulse
photocoagulation versus intravitreal injections of bevacizumab in
the treatment of central serous chorioretinopathy. Eye.

[74]  Troutbeck R, Al-Qureshi S, Guymer RH (2012). Therapeutic targeting of
the complement system in age-related macular degeneration: a review. Clin Exp Ophthalmol.

[75]  Sivaprasad S, Sandu R, Tandon A (2007). Subthreshold micropulse
diode laser photocoagulation for clinically significant diabetic
macular oedema: A three-year follow up. Clin Exp Ophthalmol.

[76]  Sivaprasad S, Elagouz M, McHugh D, Shona O, Dorin G (2010). Micropulsed
diode laser therapy: evolution and clinical applications. Surv
Ophthalmol.

